# CO_2_ Selective Potentiometric Sensor in Thick-film Technology

**DOI:** 10.3390/s8084774

**Published:** 2008-08-19

**Authors:** Kathy Sahner, Anne Schulz, Jaroslaw Kita, Rotraut Merkle, Joachim Maier, Ralf Moos

**Affiliations:** 1 Functional Materials Laboratory, University of Bayreuth, 95440 Bayreuth, Germany; 2 Max-Planck-Institut für Festkörperforschung, Stuttgart, Germany

**Keywords:** Ion conductor, solid-state reference, electrochemical sensor

## Abstract

A potentiometric sensor device based on screen-printed Nasicon films was investigated. In order to transfer the promising sensor concept of an open sodium titanate reference to thick film technology, “sodium-rich” and “sodium-poor” formulations were compared. While the “sodium-rich” composition was found to react with the ion conducting Nasicon during thermal treatment, the “sodium-poor” reference mixture was identified as an appropriate reference composition. Screen-printed sensor devices were prepared and tested with respect to CO_2_ response, reproducibility, and cross-interference of oxygen. Excellent agreement with the theory was observed. With the integration of a screen-printed heater, sensor elements were operated actively heated in a cold gas stream.

## 1. Introduction

Due to its strong impact as a greenhouse gas, monitoring of CO_2_ emissions has become crucial. Although optical detection of CO_2_ using infrared radiation is very exact, a more cost-effective method capable of also working in harsh and dirty environments is needed. To meet these requirements, several potentiometric sensor devices based on electrochemical cells with sodium conducting solid electrolytes such as β‘’-Al_2_O_3_ or Na_1+x_Zr_2_P_3-x_Si_x_O_12_ (Nasicon, 0 ≤ *x* ≤ 3) have been investigated (for a detailed review, cf. [1]). Corresponding to a “type III” electrochemical gas sensor [2], these sensor devices rely on the presence of an auxiliary phase such as sodium or barium carbonate, which is deposited at the working electrode and interacts with CO_2_. As the counter or reference electrode, gold or platinum are often used. Despite their frequent use, it has to be emphasized that such pure metal electrodes are not able to provide a thermodynamically well-defined chemical potential of sodium.

In many of these electrochemical cells, the electrolyte material forms a thin ceramic pellet or tube [3 - 8]. More recently, sensor designs based on screen-printed or dip-coated ion-conducting films have been reported [9 -14].

As a major drawback, the devices relying *only* on a carbonate auxiliary phase exhibit a pronounced cross-sensitivity towards oxygen, poor reproducibility between single sensor elements, and poor long- term stability. As an alternative approach, sodium titanate (Na_2_Ti_6_O_13_/Na_2_Ti_3_O_7_) or sodium titanate/titania (Na_2_Ti_6_O_13_/TiO_2_) mixtures were proposed as a reference system [15 - 17]. These two phase mixture systems provided a thermodynamically well-defined signal based on an oxygen-independent overall reaction as shown below.

$$\begin{array}{ll} \text{sensing electrode}: & {\text{Na}}_{2}{\text{CO}}_{3}↔2{\text{Na}}^{+}+{\text{CO}}_{2}+0.5{\text{O}}_{2}+2{\text{e}}^{-} \end{array}$$

(1) $$\begin{array}{ll} \text{reference}\;1: & 2{\text{Na}}^{+}+{\text{Na}}_{2}{\text{Ti}}_{6}{\text{O}}_{13}+0.5\;{\text{O}}_{2}+2\;{\text{e}}^{-}↔2{\text{Na}}_{2}{\text{Ti}}_{3}{\text{O}}_{7}\\ \text{overall reaction}: & {\text{Na}}_{2}{\text{CO}}_{3}+{\text{Na}}_{2}{\text{Ti}}_{6}{\text{O}}_{13}↔{\text{CO}}_{2}+2\;{\text{Na}}_{2}{\text{Ti}}_{3}{\text{O}}_{7} \end{array}$$

(2) $$\begin{array}{ll} \text{Or reference}\;2: & 2\;{\text{Na}}^{+}+6\;{\text{TiO}}_{2}+0.5\;{\text{O}}_{2}+2\;{\text{e}}^{-}↔{\text{Na}}_{2}{\text{Ti}}_{6}{\text{O}}_{13}\\ \text{overall reaction}: & {\text{Na}}_{2}{\text{CO}}_{3}+6\;{\text{TiO}}_{2}↔{\text{CO}}_{2}+{\text{Na}}_{2}{\text{Ti}}_{6}{\text{O}}_{13} \end{array}$$

As derived in detail in [15], the resulting electromotive force *emf* of these cells is related to the chemical potential difference of the sodium ions at the electrodes according to Eq. 3.

(3) $$\text{emf}=-\frac{1}{F}\left(\mu_{{\text{Na}}^{+}}^{\text{reference}}-\mu_{{\text{Na}}^{+}}^{\text{carbonate}}\right)$$

where *F* denotes the Faraday constant, and $\mu_{{\text{Na}}^{+}}^{\text{i}}$ is the chemical potential of the sodium ions at the reference and the working electrode, respectively.

Since both $\mu_{{\text{Na}}^{+}}^{\text{reference}}$ and $\mu_{{\text{Na}}^{+}}^{\text{carbonate}}$ are well-defined within this set-up, the *emf* of these cells was shown to depend solely on the carbon dioxide partial pressure *pCO_2_* and on the operating temperature *T* of the device [15]. As a consequence, simultaneous knowledge of the sensor temperature and the geometry-independent parameter *emf* enables one to precisely determine *pCO_2_*. The fact that the *emf* is defined thermodynamically also implies the absence of long-term drift effects.

Since the cells discussed in [15] were prepared from bulky ceramic pellets, their usefulness in real world applications was limited. In particular, homogeneous heating of such cells can only be achieved in a furnace, and the integration of a temperature sensor to precisely monitor the operating temperature is not straight-forward. It is therefore highly desirable to transfer the present promising sensor concept to thick-film technology, which provides a basis for miniaturization and integration of further functionalities such as heater and temperature sensor with a single sensor chip.

In this contribution, we report results obtained on long-term stable CO_2_ sensors with a sodium titanate reference prepared entirely via a cost-effective screen-printing technique. In a detailed study, the most appropriate reference system was identified. In addition to the basic sensor characteristics, the devices were tested with respect to cross-interference of oxygen and long-term stability.

## 2. Experimental

### 2.1 Precursor preparation

All ceramic precursor powders were prepared by a conventional mixed-oxide route. To obtain the *Nasicon* composition Na_1+x_Zr_2_P_3-x_Si_x_O_12_ with x = 2.2, Na_2_CO_3_ (Merck), NH_4_H_2_PO_4_ (VWR), SiO_2_ (VWR), and ZrO_2_ (AlfaAesar) were mixed in stoichiometric amounts in a ball mill and calcined at 1050 °C for 12 h.

In the case of the *sodium titanate compositions* Na_2_Ti_6_O_13_ and Na_2_Ti_3_O_7_, Na_2_CO_3_ (p.a., VWR) and TiO_2_ (anatase, Sigma Aldrich) served as precursors. The corresponding Na_2_CO_3_/TiO_2_ powder mixtures (molar ratio 1:3 and 1:6, respectively) were mixed for 4 h in a ball mill and then calcined at 900 °C for 6 h.

**Figure 1.**
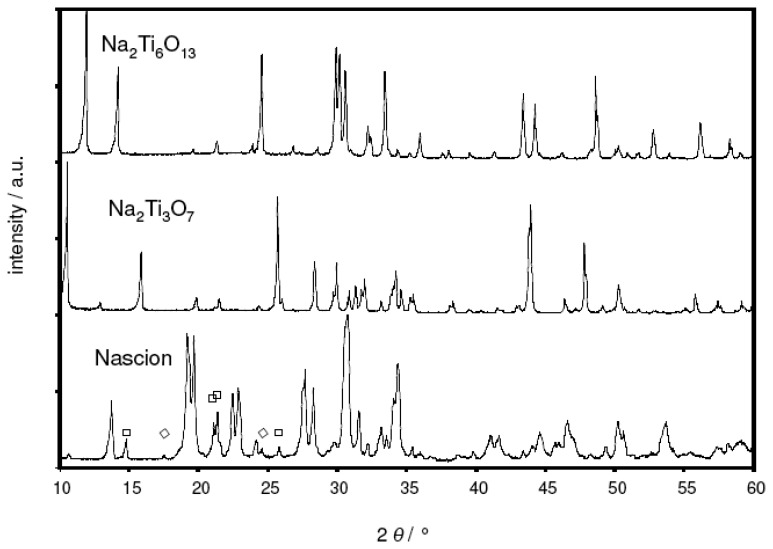
X-ray diffractograms of the as-prepared Na_2_Ti_3_O_7_, Na_2_Ti_6_O_13_ and Nasicon powder.

Figure 1 presents the XRD patterns of the as-prepared Nasicon, Na_2_Ti_3_O_7_, and Na_2_Ti_6_O_13_ powders (Philips PW 3710, Cu-K_α_ radiation, Bragg-Brentano geometry). While both sodium titanate compositions were found to be phase-pure, some impurity peaks attributed to ZrO_2_ (symbol ◊) and Na_2_ZrSi_2_O_7_ (symbol □) were observed within the Nasicon.

Two *reference compositions* were tested in the present study. The “sodium-rich” reference consisted of a Na_2_Ti_6_O_13_/Na_2_Ti_3_O_7_ (molar ratio: 1:1) mixture, while the “sodium-poor” composition was formed by mixing Na_2_Ti_6_O_13_ and TiO_2_ powder with a molar ratio of 1:1.

An eutectic mixture of Na_2_CO_3_/BaCO_3_ with a molar ratio of 1.72:1 served as the *auxiliary carbonate phase*. Prior to mixing, both precursor powders [Na_2_CO_3_ (p.a., VWR) and BaCO_3_ (Selectipur, Merck)] were dried at 450 °C. The carbonate mixture was heated at 5 K/min to 720 °C, a temperature above the melting point. The melt was retrieved from the furnace and quenched onto a brass plate. The as-obtained material was ground in a mortar.

In order to prepare screen-printable pastes, each of the as-prepared powders was sieved (< 200 µm) and mixed with a thixotropic organic binder to form a homogeneous paste.

### 2.2 Sensor preparation

**Figure 2.**
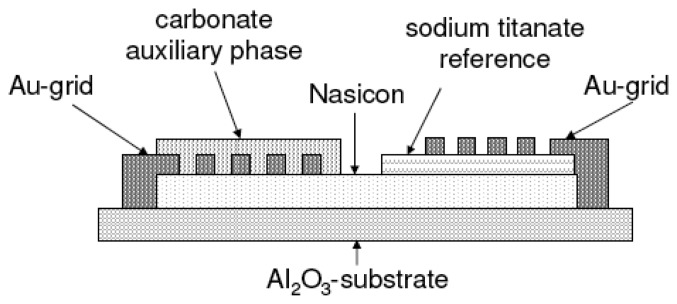
Diagrammatical representation of the sensor cross section.

The sensor set-up is shown diagrammatically in Figure 2. On top of a bare alumina substrate, a Nasicon layer was screen-printed and fired at 1050 °C for 5 h. Then, either the Na_2_Ti_3_O_7_/ Na_2_Ti_6_O_13_ or the Na_2_Ti_6_O_13_/TiO_2_ reference was printed on one side of the Nasicon film and fired at 950 °C for 5 h. Two gold grid electrodes were printed according to Figure 2 (firing at 850 °C). Finally, the sensitive Na_2_CO_3_/BaCO_3_ mixture was painted on top of one gold grid and heat treated at 600 °C.

### 2.3. XRD study

In order to study compatibility between the reference materials and the solid electrolyte, XRD studies were conducted in the 2θ range from 10 ° to 90 °. Two samples were prepared by mixing either 0.75 g Na_2_Ti_3_O_7_ with 1 g Nasicon or 0.6 g Na_2_Ti_6_O_13_ with 1 g Nasicon, respectively. While one part of these mixtures was studied directly by XRD, one part was heat-treated at 950 °C for 5 h using the sintering profile of the reference thick films.

### 2.4 Sensor tests

For tests of the sensor performance, a custom-build test bench similar to the one described in [18] for hydrocarbon sensing was used. The sensors were inserted into a tube furnace and heated to their operating temperature either with the furnace or actively via the platinum heater (see below). The total gas flow was adjusted to 200 sccm/min with dry air serving as the carrier gas. The carbon dioxide partial pressure was varied in the range of 0.4 mbar to 45 mbar by diluting pure CO_2_ gas with dry air using mass flow controllers. The actual CO_2_ concentration was monitored by an FTIR (Antaris, ThermoElectron) located downstream the sensor chamber. The *emf* output of the sensor element was monitored using a digital multimeter (Keithley 2700).

## 3. Results and Discussion

### 3.1 Sodium-rich reference

**Figure 3.**
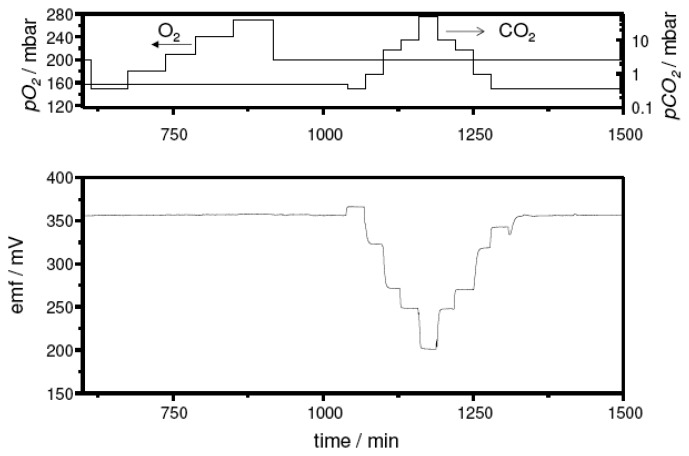
Sensor response towards *pCO_2_* and oxygen cross-interference test at 500 °C on a sensor device with the sodium-rich reference. **Top:** partial pressure of the test gases. **Bottom:** Sensor signal.

In an initial test series, sensor samples with the sodium-rich reference composition Na_2_Ti_3_O_7_/Na_2_Ti_6_O_13_ were measured. In Figure 3, the *emf* trace at 500 °C upon CO_2_ exposure is shown exemplarily. The sensor device presented a stable and perfectly reversible response. An additional variation of the oxygen partial pressure from 0.15 bar to 0.27 bar indicated no cross sensitivity of the sensor to this gas as expected from the literature.

To evaluate the sensor performance, the semilogarithmic representation *emf* = *f (*log*(pCO_2_))* was used. With the slope of this plot, the electron transfer number *n* of the electrochemical reaction can be calculated according to the Nernst equation (Eq. 4)

(4) $$\text{emf}=E_{0}-\frac{\text{RT}}{\text{nF}}\text{In}\left(\frac{{\text{pCO}}_{2}}{p^{0}}\right)=E_{0}-\frac{\mathrm{RT}\mathrm{In}10}{\underset{\text{slope}}{\underset{︸}{\text{nF}}}}\log\left(\frac{{\text{pCO}}_{2}}{p^{0}}\right)$$

with *p^0^* = 1013 mbar.

As known from the cell reactions (1) and (2), the theoretical electron transfer number equals 2. With the present sensor set up, values of 2.14 ± 0.06 (total of 4 specimens, each measured 3 times at 500 °C) and 2.12 ± 0.06 (total of 4 specimens at 600 °C) were determined.

**Figure 4.**
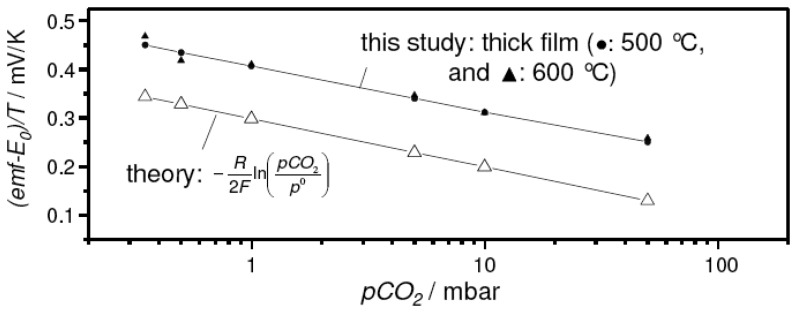
Comparison between the experimental results of the thick-film sensor with the sodium-rich reference and the results expected from theory. For details see text.

In spite of the promising sensor characteristics, the thick film devices with the sodium-rich reference presented a much higher *emf* reading than expected from the thermodynamic calculations and experimental results on the pellet-type sensor discussed in [15]. In Figure 4, this deviation between pellet-type sensor (open symbols) and the corresponding thick-film device measured in the present study (closed symbols) is presented. In this case, the temperature-corrected form of the Nernst equation (Eq. 4) was used to compare the experimental results directly with the values expected from theory.

(4a) $$\frac{\text{emf}-E_{0}}{T}=-\frac{R}{2F}\text{In}\left(\frac{{\text{pCO}}_{2}}{p^{0}}\right)$$

The required temperature-dependent values for *E_0_*, i.e. the cell *emf* at *pCO_2_* = *p^0^* = 1013 mbar, were taken from the literature [15].

**Figure 5.**
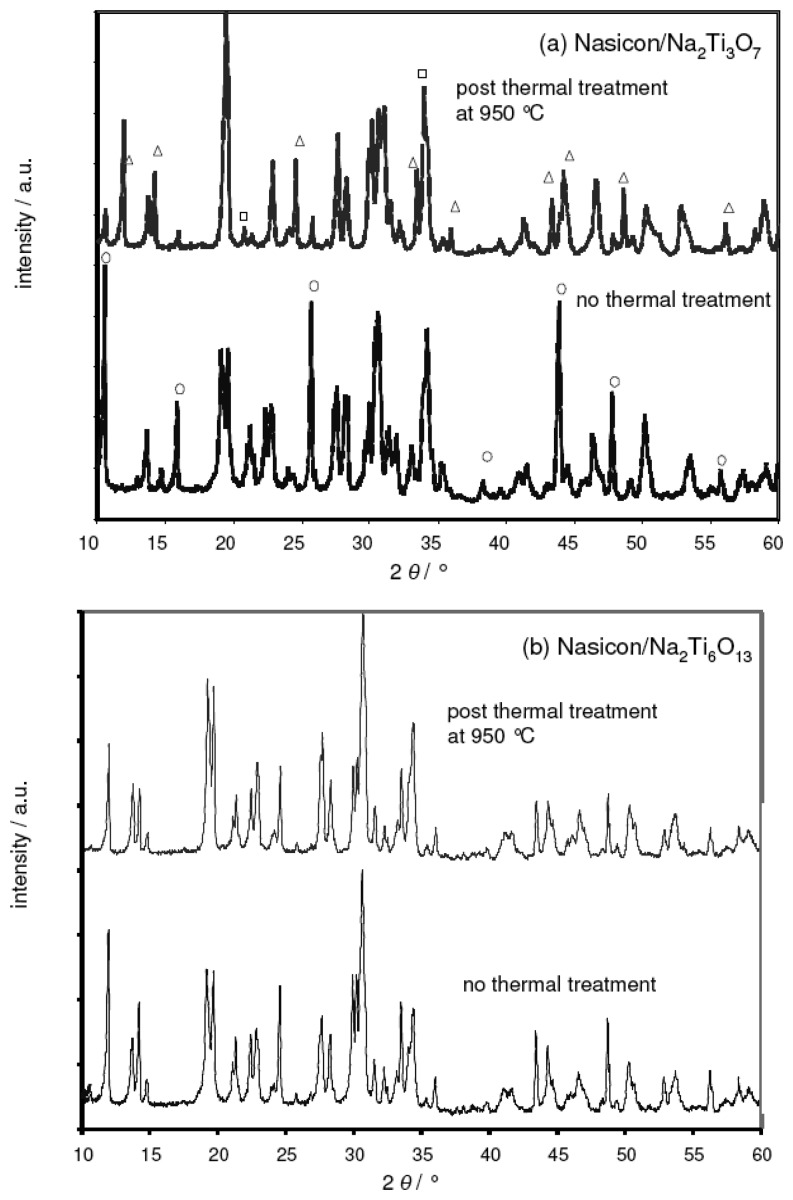
XRD pattern of Nasicon/Na_2_Ti_3_O_7_ and Nasicon/Na_2_Ti_6_O_13_ powder mixtures. **(a)** Nasicon/Na_2_Ti_3_O_7_ mixtures before (bottom) and after (top) thermal treatment at 950 °C, respectively. Only some of the characteristic peaks of the Na_2_Ti_3_O_7_ are highlighted (symbol ○), which are replaced by the sodium-poor Na_2_Ti_6_O_13_ phase (symbol &Delta:) after the thermal treatment. For details see text. **(b)** Nasicon/Na_2_Ti_6_O_13_ mixtures before (bottom) and after (top) thermal treatment at 950 °C, respectively.

In a supplementary XRD study, the deviation from the theoretical *emf* was attributed to a parasitic reaction between the Nasicon electrolyte and the Na2Ti_3_O_7_ phase. Figure 5a compares two XRD diagrams obtained from a Nasicon/Na_2_Ti_3_O_7_ powder mixture prior (bottom) and after (top) heat treatment at 950 °C, i.e., the sintering temperature of the sodium titanate reference film. For the sake of clarity, only the 2 θ range from 10° to 60° is shown. In the X-ray diffractogram of the untreated powder mixtures, the peaks from both the Nasicon and the titanate phase were identified. In addition, some impurity peaks were found, which were attributed to ZrO_2_ und Na_2_ZrSi_2_O_7_. These impurities were also present in the Nasicon precursor powder (cf. Figure 1).

The XRD pattern after thermal treatment indicated a thermally activated reaction between the Nasicon and the titanate. The characteristic peaks of Na_2_Ti_3_O_7_ (symbol ○ in the bottom figure) are almost completely replaced by the sodium-poor Na_2_Ti_6_O_13_ phase (symbol Δ). In addition, changes in the Nasicon pattern are observed, e.g., the double peak at 19° is reduced to one broadened peak. This might be attributed to a compositional and structural change of the sodium ion conductor from Nasicon to Na_4_Zr_3_Si_3_O_12_. As an additional phase, Na_3_PO_4_ is found (symbol □).

While the reactivity between Nasicon and Na_2_Ti_3_O_7_ is irrelevant in the case of the separately sintered pellets discussed in [15], it is detrimental for thick film layers that are heat-treated simultaneously. During sintering of the reference electrode, most of the Na_2_Ti_3_O_7_ phase reacts with the adjacent Nasicon layer. Due to the different phase composition of the reference electrode mixture its sodium ion activity decreases. As a consequence, the *emf* value of the cell, which is given by the difference of the chemical potentials of sodium ions at each electrode (Eq. 3), is expected to increase.

### 3.2 Sodium-poor reference

In contrast to Na_2_Ti_3_O_7_, XRD studies on Nasicon/Na_2_Ti_6_O_13_ mixtures yielded no evidence for parasitic reactions at 950 °C. Identical XRD patterns were observed prior and after thermal treatment (Figure 5b; no new peaks, only some minor changes in peak intensities appear). The sodium-poor reference composition Na_2_Ti_6_O_13_/TiO_2_ was thus identified as a more promising candidate for thick-film devices.

**Figure 6.**
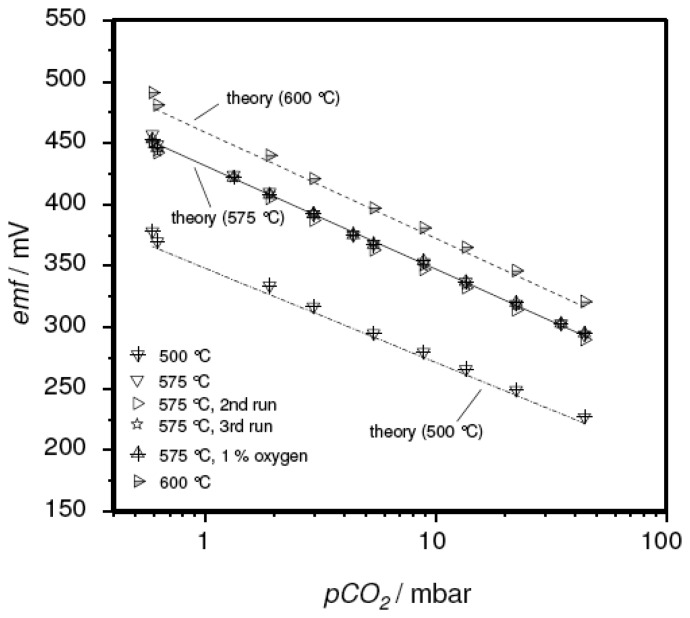
*emf* Sensor characteristics of a thick-film device with the sodium-poor reference composition. Operating temperature as indicated. For comparison, data reported in the literature for a pellet-like sensor [15] were included.

Figure 6 exemplarily presents the sensitivity plots of a corresponding sensor element. The device was measured several times at three temperatures. In contrast to the sensor devices discussed above, the measured *emf* values (symbols) were found to agree well with the results reported in the literature (lines). Again, Nernstian behavior with an electron transfer number of 1.9 was found. The reproducibility of the measurements is remarkable. In Figure 6, one can hardly distinguish the different runs at 575 °C

**Figure 7.**
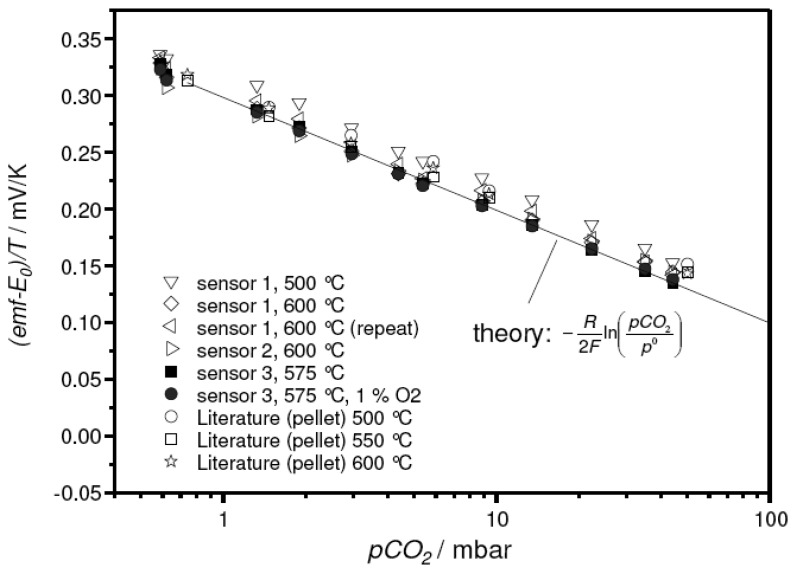
Reproducibility of the thick-film sensor concept. For comparison, data reported in the literature for a pellet-like sensor [15] were included, as well as the values expected from theory (Eq. 4a, solid line).

To ensure the reproducibility of the sensor concept, three sensor devices were prepared and measured at various temperatures. The results are summarized in Figure 7. As before (cf. Figure 4), the very sensitive representation:

$$\frac{\text{emf}-E_{0}}{T}=-\frac{R}{2F}\text{In}\left(\frac{{\text{pCO}}_{2}}{p^{0}}\right)$$

was used, in this case to emphasize the excellent agreement of the thick-film device with theory and the data on pellet-type sensors [15].

Based on these promising results, actively heated devices with the sodium-poor reference material were prepared. For this purpose, a sensor chip was glued on top of a screen-printed platinum heater using ceramic paste. Silver served as a shielding layer to prevent voltage interferences from the heater. The sensor was then mounted into a corresponding sample holder attached to a voltage source. By monitoring the resistance of the platinum heater after previous calibration, its temperature could be controlled in the range from 100 °C to 700 °C. Again, operating temperatures of 500 °C and 600 °C were chosen.

**Figure 8.**
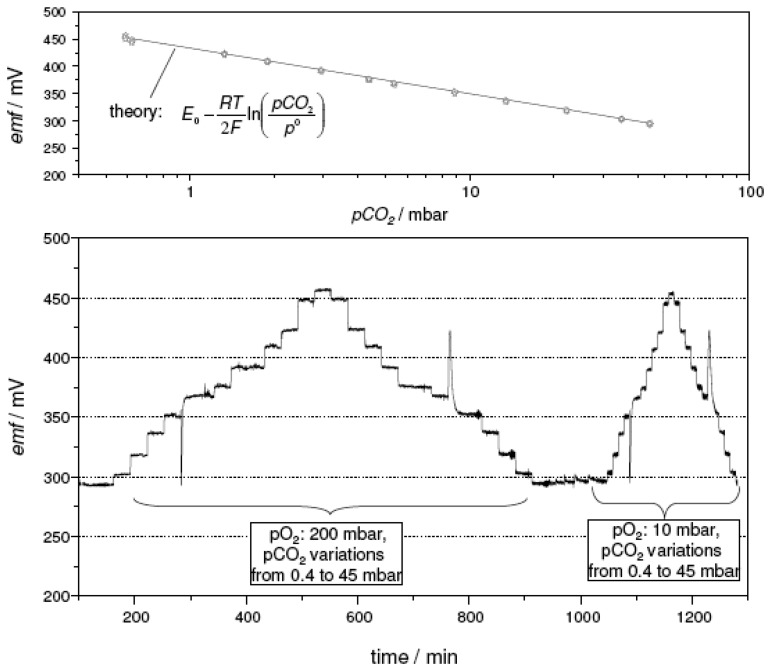
*emf* response of a thick-film device with the sodium-poor reference composition, heated to 575 °C by a screen-printed platinum heater. **Bottom part**: *emf* trace. **Top part**: semilogarithmic plot as a function of *pCO_2_* (values expected from theory (Eq. 4) were included for comparison as solid line). Total gas flow: 200 sccm/min. For details see text.

Figure 8 presents the results of four consecutive measurement cycles conducted on a thick**-**film sensor with the sodium-poor reference. The temperature of the device was adjusted to 575 °C and exposed to a cold gas stream of 200 sccm/min. The carbon dioxide partial pressure *pCO_2_* was varied stepwise between 0.4 mbar and 45 mbar. The spikes that occur consistently between the 13.5 mbar and the 9 mbar step are an artifact related to the gas dosage system of the test bench. In order to access low *pCO_2_* values, the gas dosage is switched to a dilution line and back again to achieve high *pCO_2_*. This switching is accompanied by a pressure surge leading to the observed *emf* spikes. The actively heated sensor presented a stable and reproducible response, which was insensitive to a variation in the oxygen partial pressure from 200 mbar (20 % O_2_) to 10 mbar (1 % O_2_). As shown in the top part of Figure 8, the sensor characteristics of each measurement cycle coincide. The electron transfer number calculated from the slope of this semilogarithmic plot equaled 1.94. For comparison, the values expected from theory were included in Figure 8 as a solid line. They were calculated using the Nernst equation (Eq. 4) with the *E_0_* value estimated from Ref. [15].

## 4. Conclusions

The potentiometric sensor concept with an open sodium titanate reference, which had formerly been investigated in a ceramic pellet set-up, was successfully transferred to thick-film technology. After identifying the appropriate reference composition, screen-printed sensor devices were prepared and tested with respect to CO_2_ response, reproducibility, and cross-interference of oxygen. For the thick- film sensors using a sodium-poor reference formulation, excellent agreement with the theory was observed. After attaching a screen-printed heater, sensor elements were operated actively in a cold gas stream.

Future work is directed to further miniaturizing the thick film sensor, i.e., in a hot-plate set-up as shown in [19] and [20]. In particular, the direct integration of a heating element on one single chip is envisioned.
